# Platelet-activating factor receptor (PAFR) regulates neuronal maturation and synaptic transmission during postnatal retinal development

**DOI:** 10.3389/fncel.2024.1343745

**Published:** 2024-03-20

**Authors:** Barbara Dalmaso, Andre Mauricio Passos Liber, Dora Fix Ventura, Sonia Jancar, Carolina Beltrame Del Debbio

**Affiliations:** ^1^Department of Cell and Developmental Biology, Biomedical Sciences Institute, University of São Paulo (ICB-USP), São Paulo, Brazil; ^2^Université Paris-Saclay, CNRS, Institut des Neurosciences Paris-Saclay, Saclay, France; ^3^Department of Experimental Psychology, Institute of Psychology, University of São Paulo (IP-USP), São Paulo, Brazil; ^4^Department of Immunology, Biomedical Sciences Institute, University of São Paulo (ICB-USP), São Paulo, Brazil

**Keywords:** PAF receptor, retina, retinal development, retinal neurogenesis, electroretinography

## Abstract

**Introduction:**

Platelet-activating factor (PAF), PAF receptor (PAFR), and PAF- synthesis/degradation systems are involved in essential CNS processes such as neuroblast proliferation, differentiation, migration, and synaptic modulation. The retina is an important central nervous system (CNS) tissue for visual information processing. During retinal development, the balance between Retinal Progenitor Cell (RPC) proliferation and differentiation is crucial for proper cell determination and retinogenesis. Despite its importance in retinal development, the effects of PAFR deletion on RPC dynamics are still unknown.

**Methods:**

We compared PAFR knockout mice (PAFR^−/−^) retinal postnatal development proliferation and differentiation aspects with control animals. Electrophysiological responses were analyzed by electroretinography (ERG).

**Results and discussion:**

In this study, we demonstrate that PAFR^−/−^ mice increased proliferation during postnatal retinogenesis and altered the expression of specific differentiation markers. The retinas of postnatal PAFR^−/−^ animals decreased neuronal differentiation and synaptic transmission markers, leading to differential responses to light stimuli measured by ERG. Our findings suggest that PAFR signaling plays a critical role in regulating postnatal RPC cell differentiation dynamics during retinal development, cell organization, and neuronal circuitry formation.

## 1 Introduction

Retinal development is a rigorously coordinated process in which RPCs generate different neurons in a temporal sequence conserved among vertebrates (Alexiades and Cepko, 1996; Miesfeld and Brown, 2019). Retinal neurogenesis initiates with RPCs differentiating in waves into ganglion cells (RGC), horizontal cells, amacrine cells, and cone photoreceptors, marking the early retinogenesis phase. The early retinogenesis overlaps with the beginning of the late retinogenesis with the generation of most of the rod photoreceptors, bipolar, and Müller cells (Alexiades and Cepko, 1996; Hoon et al., 2014; Hoshino et al., 2017).

It is well known that RPC differentiation and neuronal fate are regulated by intrinsic signals, including those related to proliferation, stemness maintenance, cell fate commitment, final differentiation, and cell specialization (Alexiades and Cepko, 1996; Miles and Tropepe, 2016). The transcriptional profiling during retinal neurogenesis indicated an opposite correlation between cell cycle dynamics and the expression of differentiation-specific genes (Blackshaw et al., 2004; Barton and Levine, 2008). The RPC decision of cell cycle reentry or exit after the final mitosis is very important to promote the correct retinal layer formation and maturation and the correct amount of each cell type within the retina. Cell cycle controllers are closely regulated during retinogenesis. Repressed cell cycle reentry through cyclin deficiency leads to an impaired neuronal maturation profile and defective retinal tissue structuring (Fantl et al., 1995; Das et al., 2009). Prolonged cell cycle by deletion of cell cycle controllers such as p27kip1 and p19Ink4d induces changes in the arrangement of retinal layers and differences in the proportion of cell types (Cunningham et al., 2002; Lanctot et al., 2017). Thus, the balancing ratio between proliferation and differentiation was revealed to be crucial for proper retinogenesis.

It is well established that RPC undergoes a series of cell fate determinations before and after the final mitosis. However, the mechanisms involved in cell fate decisions and differentiation paths re not yet completely clear. Among many factors that could influence cell fate decision and proper differentiation, the bioactive lipid platelet-activating factor (PAF) may play critical roles in regulating cell proliferation and neuronal maturation processes in a wide range of CNS tissue, including the retina (Kumar et al., 1988; Goracci et al., 2009; Dalmaso et al., 2020).

PAF is an important inflammatory lipid mediator produced by lyso-PAF acetyltransferases (LPCATs) and catabolized to its inactive form by PAF-acetyl hydrolases (PAF-AHs) (Livnat et al., 2010). PAF molecule biogenesis is regulated by different cellular stimuli and acts as an autocrine and/or paracrine second messenger binding to its receptor (PAFR) (Maclennan et al., 1996; Harayama et al., 2008). PAFR is a pleiotropic G-protein-coupled receptor expressed in the plasma and nuclear cellular membrane of several CNS and retinal cell types, such as neurons, microglia, and astrocytes (Bazan et al., 1994; Mori et al., 1997). It is well known that PAF/PAFR activation is upstream of important regulatory mechanisms of cell proliferation, migration, inflammation, and apoptosis, such as the MAPK/ERK pathway, PI3K, Jak2, and NFkB (Hwang and Lam, 1986; Bernatchez et al., 2001; Honda et al., 2002).

PAF biogenesis machinery is present in the developing retina (Bussolino et al., 1989; Fragel-Madeira et al., 2011). PAF levels are downregulated in the early stages of retinogenesis by the presence of high amounts of PAF-AH. Later, a shift in PAF production during retinal differentiation is detected after a gradual increase in LPCAT expression (Bussolino et al., 1988; Finnegan et al., 2008). It suggests that PAF and PAF-related enzymes are important in RPC differentiation. Indeed, previous studies demonstrated that RPC treated with PAF became arrested in the S/G2 cell cycle phase transition, reducing nuclear interkinetic migration and retinal progenitors' proliferation due to p21^cip1/waf1^ and cyclin B1 regulation (Fragel-Madeira et al., 2011; Damiani et al., 2017). PAFR stimulation in primary embryonic rat neuron cultures induced precocious development of axon-like extensions and a concentration-dependent increase in neuronal-specific enzyme activities (Kornecki and Ehrlich, 1988; Ved et al., 1991). PAF is also associated with the production and release of the neurotransmitters acetylcholine (ACh), glutamate, and dopamine (Bussolino et al., 1989; Dinday et al., 2017).

Although PAF/PAFR may play important roles in regulating neural progenitor proliferation and differentiation processes, the effects of PAFR ablation on developing RPC dynamics are still unknown. In this study, we determined PAFR, PAFAH, and LPCAT expression in the mammalian postnatal retinas. We compared the retinas of wild-type (WT) mice with the retinas of PAFR-null (PAFR^−/−^) animals at three different time points: postnatal day 1 (PN1), containing cells differentiating during late retinogenesis at PN10, which marks the end of the late retinogenesis stage, and after complete differentiation of the retinas at PN30. We show a substantial modulation of PAFR and PAF-related enzymes during retinal development, with increased PAFR during retinal differentiation. The deletion of PAFR resulted in increased expression of proliferation markers and altered the expression of neural development and differentiated neuron markers. These changes resulted in a decrease in synaptic transmission machinery and electrophysiological responses. Our data suggest that PAFR signaling could be important in regulating RPC proliferation and neuronal differentiation.

## 2 Materials and methods

### 2.1 Experimental model/animals

PAF receptor knockout mice (PAFR^−/−^), with deletion of the *Ptafr* gene (BRC No. RBRC01733; Strain B6.129P2-Ptafr), were previously described (Ishii and Shimizu, 2000). Postnatal animals with 1, 10, and 30 days, along with age-matched wild-type C57Bl6/J mice, were housed in the Department of Cell and Developmental Biology's Animal Facility at the University of Sao Paulo, Brazil. The animals were maintained under 12-h light/dark cycles with *ad libitum* access to water and food. All experimental procedures followed the guidelines adopted by the Brazilian Society of Sciences in Laboratory Animals (SBCAL) and were approved by the ethical committee for Animal Research of the Institute of Biomedical Sciences of the University of Sao Paulo (protocol number #3588090419).

### 2.2 Transcriptional expression analysis

The total RNA was isolated with the TRIzol method (Thermo Fisher Scientific, MA, USA), and cDNA (1 ug/uL) was synthesized using SuperScript™ Reverse Transcriptase reagent (Thermo Fisher Scientific, MA, USA). Gene expression was analyzed by quantitative polymerase chain reaction with the Quantifast SYBR Green PCR Kit (Qiagen, HI, Germany) and the QuantStudio 3 Real-Time PCR System (Thermo Fisher Scientific, MA, USA). Specific primers (Table 1) were amplified and normalized using the Q-Gene software method, as previously described (Muller et al., 2002). β*-Actin* was applied as a housekeeping gene.

**Table 1:** List of specific primers.

**Gene**	**Sequence**
*Ptafr (Pafr)*	F: 5′-AGCAGAGTTGGGCTACCAGA-3′ R: 5′-TGCGCATGCTGTAAAACTTC-3′
*Lpcat2*	F: 5′-CCAGGTGGCATTTAAGCTCT-3′ R: 5′-TCTTGGCATATTCTGGGTGC-3′
*Pafah*	F: 5′-GTCTCTGCTTCAGAGGATGC-3′ R: 5′-ACATTGTGATCGTGACCGTG-3′
*Neurod1*	F: 5′-ACGCAGAAGGCAAGGTGTCC-3′ R: 5′-TTGGTCATGTTTCCACTTCC-3′
*Tubb3*	F: 5′-GCTGTCCGCCTGCCTTTT-3′ R: 5′-GACCTCCCAGAACTTGGCC-3′
*Map2*	F: 5′-CCGGGTAGATCACGGGGCTG-3′ R: 5′-GTCGTCGGGGTGATGCCACG-3′
*Rbfox3 (NeuN)*	F: 5′-CACCACTCTCTTGTCCGTTTGC-3′ R: 5′-GGCTGAGCATATCTGTAAGCTGC-3′
*Ops1*	F: 5′-ACTCAGCATCATCGTGCTCTGCTA-3′ R: 5′-AGTATGCGAAGACCATCACCACCA-3′
*Rho*	F: 5′-TGCCACACTTGGAGGTGAAA-3′ R: 5′-ACCACGTAGCGCTCAATGG-3′
*Calb1 (Calbindin)*	F: 5′-GTGCTTTGGGTGACAGTCCT-3′ R: 5′-TGAGCTGGATGCTTTGCTGA-3′
*Calb2 (Calretinin)*	F: 5′-ATGGAAGCGGCTATATTGATGAGA-3′ R: 5′-TCGGCCAAGGACATGACAC-3′
*Chat*	F: 5′-GAGCGAATCGTTGGTATGACAA-3′ R: 5′-AGGACGATGCCATCAAAAGG-3′
*Syp*	F: 5′-ACTTCAGGACTCAACACCTCGG-3′ R: 5′-GAACCATAGGTTGCCAACCCAG-3′
*Actb (β-Actin)*	F: 5′-TGAGCTGCGTTTTACACCCT-3′ R: 5′-GCCTTCACCGTTCCAGTTTT-3′

### 2.3 Immunohistochemistry

For each animal background (WT and PAFR^−/−^) and different age groups investigated (PN1, PN10, and PN30), the eyes of four to five different animals were collected. The eyes were enucleated and fixed in a 4% paraformaldehyde (PFA) solution for 30 min at room temperature (RT) and then transferred to 30% sucrose overnight for cryoprotection. Eyes were embedded in the OCT solution and sectioned in cryostat. Retinal sections were incubated with a blocking solution (1% bovine albumin, 10% animal serum, and 0.3% PB/Triton X-100) for 60 min at RT and proceeded to incubation of primary antibodies for 12–16 h. Next, samples were washed in a saline solution and incubated for 2 h at room temperature with specific secondary antibodies (Alexa Fluor 555 or Alexa Fluor 488, Thermo Fisher). Primary antibodies used in this study include rabbit anti-PAFR 1:100 (Cayman Chemical), mouse anti-Calbindin 1:200 (Sigma-Aldrich), rabbit anti-Calretinin 1:200 (Chemicon), rabbit anti-Rho 1:200 (Millipore), and mouse anti-Sws1 1:200 (Chemicon) antibodies. Images were obtained with a CCD camera attached to a fluorescence microscope and analyzed using ImageJ software. The manipulation of the images was restricted to threshold and brightness adjustments to the whole image. Controls for the experiments consisted of omitting primary antibodies; no staining was observed in these cases. Nuclei were counterstained with 4′,6-diamidine-2′-phenylindole dihydrochloride (DAPI; Sigma-Aldrich).

### 2.4 Western blotting

For each animal background (WT and PAFR–/–) and different age groups investigated (PN1, PN10, and PN30), we collected and pooled the retinas from two animals (four retinas). Each pool of retinas was considered as one independent sample. We analyzed four independent samples (*N* = 4) per background and age. Independent samples were immersed in ice-cold 20 mM Tris/HCl (pH 8.0) with protease inhibitors (0.4 mM phenylmethylsulfonyl fluoride, 20 μM leupeptin, 0.005 trypsin inhibiting U/ml aprotinin, and 2 μg/ml soybean trypsin inhibitor) and homogenized. Cell debris was discarded after centrifugation (15,000 *g* for 15 min at 4°C). Protein was determined with a Bradford assay (Bio-Rad Laboratories, MA, United States), and 50 μg of protein was applied to 12% SDS-PAGE for electrophoresis and later transferred to nitrocellulose membranes. Non-specific binding sites were blocked with 5% bovine serum albumin (BSA) in TBS-T buffer (150 mM NaCl, 20 mM Tris, 0.5% Tween 20, pH 7.4) for 1 h. Membranes were incubated overnight with primary antibodies, followed by 2 h of incubation with specific secondary peroxidase-conjugated antibodies (1:2,000). Detection of labeled proteins was achieved using SuperSignal West Pico Chemiluminescent Substrate (Thermo Fisher Scientific, MA, United States) and obtained with the imaging system GBox Chemi XX6 and GeneSys Software (Syngene, KA, India). Densitometric analysis was performed using ImageJ imaging software and represented as an arbitrary unit (AU), with β-actin as an endogenous control. Primary antibodies used in this study include rabbit anti-PAFR 1:200 (Cayman Chemical), mouse anti-Cyclin A2 1:1,000 (Invitrogen), rabbit anti-β tubulin III 1:2,000 (Sigma-Aldrich), goat anti-Chat 1:500 (Sigma-Aldrich), mouse anti-β actin 1:2,000 (Invitrogen), and mouse anti-NeuN 1:500 (Millipore) antibodies.

### 2.5 Gene expression datasets

The eyeIntegration transcriptome database is available in the public domain at https://eyeIntegration.nei.nih.gov (Bryan et al., 2018; Swamy and McGaughey, 2019), and human adult and fetal retina datasets were collected (Li et al., 2014; Whitmore et al., 2014; Mustafi et al., 2016; Aldiri et al., 2017; Hoshino et al., 2017; Mellough et al., 2019). Downloaded data were given in transcripts per million and then normalized in log_2_. We compared PAFR (PTAFR), LPCAT2, and PAFAH1 expression at early retinal development (fetal 52–59 days old retinas), late retinal development (fetal 107–161 days old retinas), and adult retinas.

### 2.6 Electroretinography

C57Bl6/J WT (*n* = 12) and PAFR^−/−^ (*n* = 15) mice were transported to the Institute of Psychology (IP) at USP. They were kept for at least 1 week to acclimatize to the new environment, with 12-h light/dark cycles and *ad libitum* access to water and food to avoid possible changes in ERG recordings caused by stress. The protocol was an extended version of the ISCEV protocol (International Society for Clinical Electrophysiology of Vision). Protocols and procedures have been described in detail elsewhere (Tsai et al., 2016; Barboni et al., 2020). Briefly, before the ERG recordings, mice were dark-adapted for at least 12 h. Animal handling, preparation, and electrode placement were performed under deep red illumination to keep the retina dark-adapted. The animals were anesthetized with an intramuscular injection of 2% xylazine hydrochloride (Calmium, Agener) and 10% ketamine hydrochloride (Ketamine, Agener) (1:1, 1 μl/g of animal). During the recordings, the mice were positioned on a water-heated platform (38°C) to maintain body temperature during anesthesia. To prevent dehydration, we subcutaneously injected 0.9% saline before (300 μl) and after (100 μl) recordings. Pupils were fully dilated with 1% tropicamide (Mydriacyl^®^ Alcon) and 10% phenylephrine (Allergan) eye drops, followed by topical anesthesia with 0.5% proxymetacaine (Anestalcon; Alcon) to avoid any corneal discomfort. Goldring electrodes (Ø 1 mm; Roland Consult, Brandenburg, Germany) were used as active electrodes, positioned on the corneas with methylcellulose 2% (Ophthalmos, São Paulo, Brazil). To protect and prevent corneal dehydration, methylcellulose was applied after recordings. Two needle electrodes were placed subcutaneously medial to the ears (reference electrodes), and one was positioned subcutaneously at the base of the tail (ground electrode) (Concentric Subdermal Steel Needle; Roland Consult, Brandenburg, Germany). Binocular recordings of full-field ERGs and stimulus presentations were performed using the RetiPort system (Roland Consult, Brandenburg, Germany) with a Ganzfeld bowl (Q450SC, Roland Consult). Signals were amplified 100,000 times, filtered with a bandpass filter between 1 and 300 Hz, and digitized at a rate of 512 (flashes) or 1,024 Hz (flicker). Data were analyzed offline by peak/trough detection and Fourier analysis using Matlab^®^ (The Mathworks Inc., Natick, MA, United States) and Excel (Microsoft Office 2016, ©Microsoft Corporation, Redmond, WA, United States). The oscillatory potentials (OPs) were isolated by a variable filter method, and ERGs without OPs were used to measure a- and b-wave parameters. Isolated OPs (2, 3, and 4) were also analyzed. The scotopic a-wave amplitude was defined as the difference in μV between the baseline and the trough after stimulus onset. The scotopic b-wave amplitude was the difference in μV between the a-wave trough and the b-wave peak. The implicit times corresponded to the intervals between the stimulus onset, the a-wave trough, and the b-wave peak. OPs were analyzed in the time domain following the same criteria. The b-waves of the light-adapted flash ERG were measured as described for scotopic recordings. As previously shown, the photopic a-wave components were considerably reduced and were not included in the analyses (Tsai et al., 2016; Barboni et al., 2020). The On/Off recordings and Flicker ERGs (sine-wave modulation) were Fourier analyzed to obtain the amplitudes and phases of the first harmonic. ERGs were recorded in order of increasing mean luminance with different protocols, as described in detail elsewhere (Tsai et al., 2016; Barboni et al., 2020). In summary, the following protocols have been recorded: (1) *Scotopic flashes*: Dark-adapted rod and mixed rod-cone-mediated ERG responses were recorded to flashes (white light) of −3.7, −2.7, −1.7, −0.7, and 0.3 log cd.s/m^2^ strengths on a dark background; (2) *Meospic On- and Off-responses:* Rapid-On and Rapid-Off sawtooth stimuli (white light) were presented at 4 Hz with 100% luminance (Michelson) contrast at a mean luminance of 1 cd/m^2^, evoking On- (to luminance increments) and Off- (to luminance decrements) responses; (3) *Photopic flashes*: White flashes of 0.3 log cd.s/m^2^ strength were taken on a white background of 25 cd/m^2^; (4) *Photopic sine-wave:* sinusoidal luminance modulation (100% Michelson contrast; 60 cd/m^2^ mean luminance—white light) were measured randomly at 10 temporal frequencies between 3 and 30 Hz; and (5) *Photopic On- and Off-responses*: Rapid-On and Rapid-Off sawtooth stimuli (white light) evoking On- (to luminance increments) and Off-responses (to luminance decrements) were obtained at a mean luminance of 60 cd/m^2^ at 4 Hz with 100% luminance (Michelson) contrast as in the mesopic condition.

### 2.7 Statistical analysis

For PCR and WB experiments, the retinas of each animal background (WT and PAFR^−/−^) and different age groups (PN1, PN10, and PN30) were collected and pooled. Each pool of retinas was considered an independent sample. For PCR, we analyzed five independent samples containing two pooled retinas in each sample (*N* = 5). For WB, we analyzed four independent samples containing four pooled retinas in each sample (*N* = 4). For immunohistochemistry experiments, we analyzed four to five retinas. Statistical differences were calculated by an unpaired Student's *t*-test (two-tailed) or a two-way ANOVA with a multiple comparison test (three or more conditions) through comparative analysis between treatments and the respective controls using GraphPad Prism Software (GraphPad, Inc., San Diego, CA, United States). Normalization was performed with β-actin (gene and protein). The results were expressed as the mean ± SEM (standard error of the mean). The data were presented in Arbitrary Units (AU). For the ERG data, we analyzed 24 and 30 eyes for wild-type and PAFR^−/−^ mice, respectively. The eyes that presented aberrant measurements, either for amplitude or implicit time/phase, were removed from the sample, taking into account the following calculation: LI – 2 × 1.5 × (LS – LI) and LS + 2 × 1.5 × (LS – LI), where LI is the lower limit and LS is the upper limit, which corresponds to the standard deviation values. Statistical analysis of ERG data was performed with the Kruskal-Wallis two-tailed test with multiple comparisons using the Statistica 10.0 software (StatSoft Inc.). All ERG data were expressed as means ± SD (standard deviation). *P-*values < 0.05 were considered statistically significant for all the analyses.

## 3 Results

### 3.1 PAF receptor and PAF-related enzymes are regulated during human and mouse retinal differentiation

**Figure 1:**
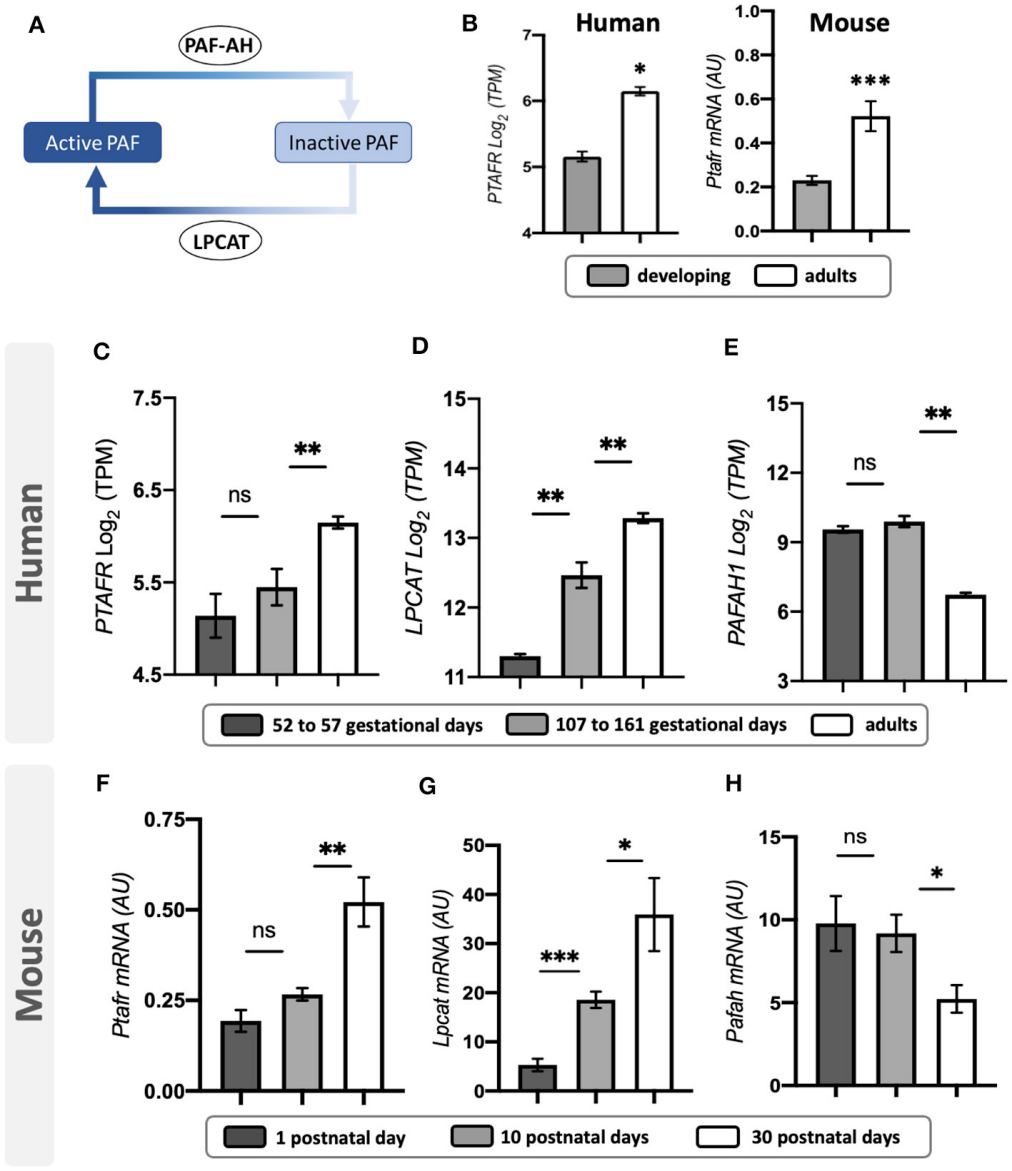
PAF pathway expression in human and mouse retinas. **(A)** Schematic representation of the PAF regulatory mechanism. The PAF molecule is activated by lyso-PAF acetyltransferase (LPCATs) and catabolized to its inactive form by PAF-acetyl hydrolases (PAF-AHs). **(B)** PAF receptor (*PTAFR*) transcriptional expression during development and fully differentiated retinas of humans and mice. **(C–E)** *PTAFR, LPCAT*, and *PAFAH1* transcriptional expression in different human fetal retina developmental phases: early development (52–57 days old, *n* = 07) and late development (107–161 days old, *n* = 14), or adult retinas (*n* = 20). **(F–H)** *PTAFR, LPCAT*, and *PAFAH1* mRNA expression during postnatal mice retinal developmental phases: postnatal (PN) day 1 (*n* = 5), day 10 (*n* = 5), and day 30 (n = 5). Results are given as mean ± SEM after normalization by log2 of transcripts per million (TPM). ^*^P < 0.05; ^**^P < 0.01; ^***^P < 0.001. ns, non-significant. Retrieved datasets from the eyeIntegration database (Swamy and McGaughey, 2019). PAF, platelet-activating factor.

To determine the expression of PAF receptor and biogenesis mechanism in the retina (Figure 1A), we first analyzed the transcriptional expression of PAF receptor (PTAFR), PAF activating enzyme (lyso-PAF acetyltransferase, LPCAT2), and PAF inactivation enzyme (PAF-acetylhydrolase, PAFAH) in human retina datasets retrieved from the eyeIntegration web database (Bryan et al., 2018; Swamy and McGaughey, 2019) and mouse retinas.

Adult human and mouse retinas express higher levels of PAFR transcripts than developing retinas (Figure 1B). During human retinal development, the expression of PAFR (Figure 1C) was not statistically different at 52–57 and 107–161 gestational days (52–57 = 5.1 ± 0.3; 107–162 = 5.4 ± 0.4). Significant overexpression of PAFR was observed in adult retinas compared to fetal time points (adult = 6.1 ± 0.2; *p* < 0.01). LPCAT indicated increased expression from retinal development stages to adults (Figure 1D). No differences were found in PAFAH expression during retinal development stages, but a significant downregulation was observed in adults (Figure 1E).

Mouse retinas presented similar expression patterns of PAF regulatory mechanisms in comparison to humans. PAFR (Figure 1F) indicated no significant modulation from 1 to 10 postnatal days (PN1 = 0.7 ± 0.05; PN10 = 0.2 ± 0.09), but increased expression in adult retinas (adult = 0.4 ± 0.1). LPCAT indicated consistently increased expression from retinal development to the adult stage (Figure 1G), and PAFAH significantly decreased in adult retinas, with no differences observed between PN1 and PN10 (Figure 1H).

**Figure 2:**
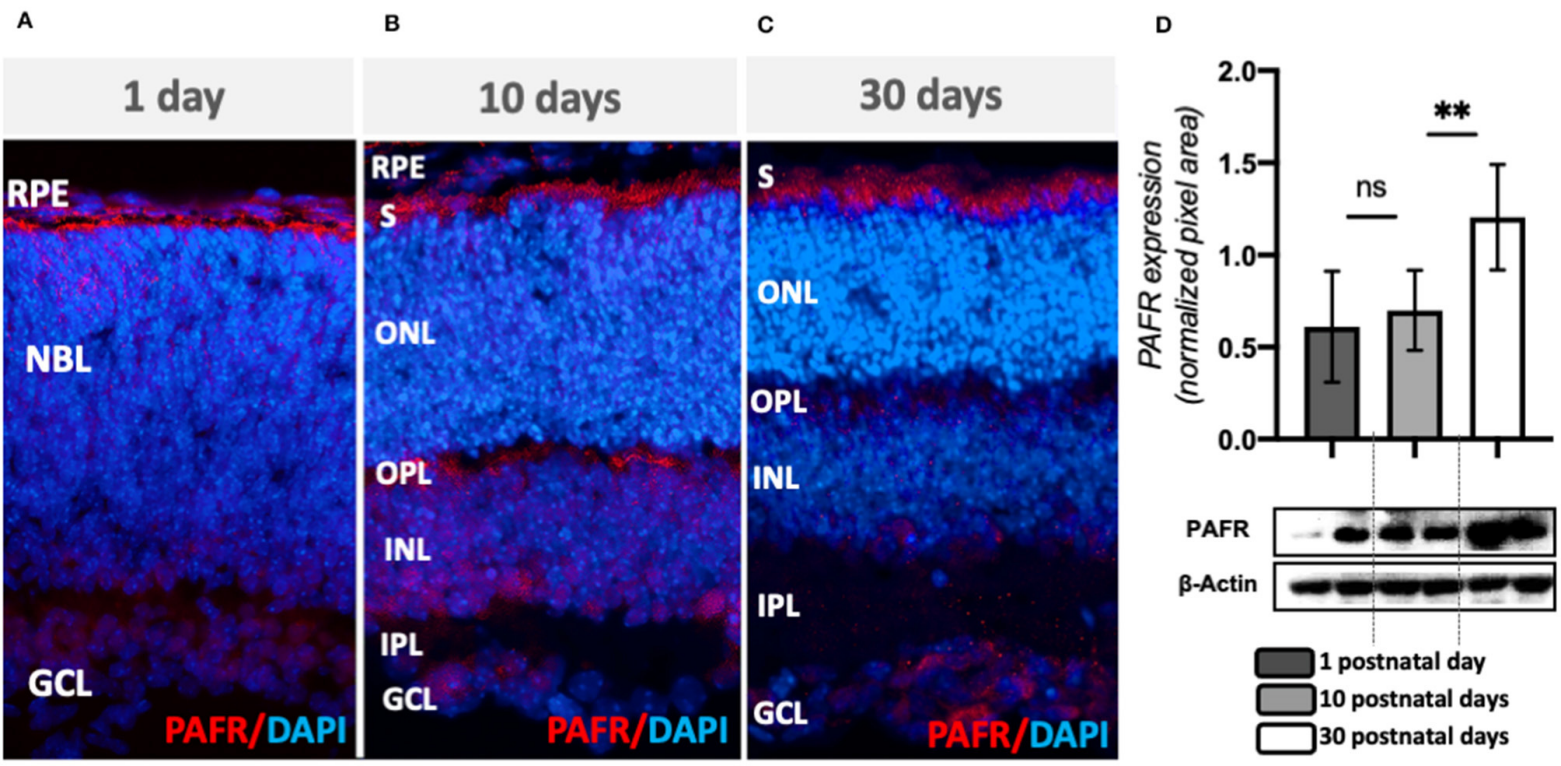
PAFR protein expression. **(A)** At postnatal day 1 (PN1), PAFR was detected at the newly formed retinal pigmented epithelium (RPE), neuroblastic layer, and retinal ganglion cell (RGC) layer (*n* = 4). **(B)** At PN10, PAFR was detected in RPE, photoreceptor segment layer (S), outer and inner plexiform layers (OPL and IPL), and at the RGC layer (*n* = 4). **(C)** In adult retinas at PN30, PAFR was detected in all retinal layers (*n* = 4). **(D)** Western blotting quantification analysis indicated no differences in expression between PN1 and PN10 but increased expression at PN30. Each band is representative of 1 independent sample, and 4 samples were analyzed per group (*n* = 4). Data are shown as mean ± S.E.M. ^**^P < 0.01. ns, non-significant.

An immunohistochemistry analysis on mouse retinas indicated PAFR expression in the neuroblastic layer (NBL), with higher expression at the future outer nuclear layer, retinal pigmented epithelial (RPE), and few RGC cells at PN1 (Figure 2A, Supplementary Figures 1A–C). At PN10, PAFR was observed in the RPE and photoreceptor segment layers, OPL, and INL (Figure 2B, Supplementary Figures 1D–F). PAFR was expressed in RPE and photoreceptor segments in adults, with low expression at plexiform layers and GCL (Figure 2C, Supplementary Figures 1G–I). Total protein quantification indicated higher expression of PAFR in adult retinas in comparison to PN1 and PN10 (Figure 2D), similar to transcriptional expression.

These results suggested that PAFR and activating enzymes increased expression during retinal postnatal differentiation and could play an important role in retinal cell specification and function in adults.

### 3.2 PAFR knockout mice (PAFR^−/−^) have differential expression and the ratio of PAF-related enzymes in adult and postnatal developing retinas

**Figure 3:**
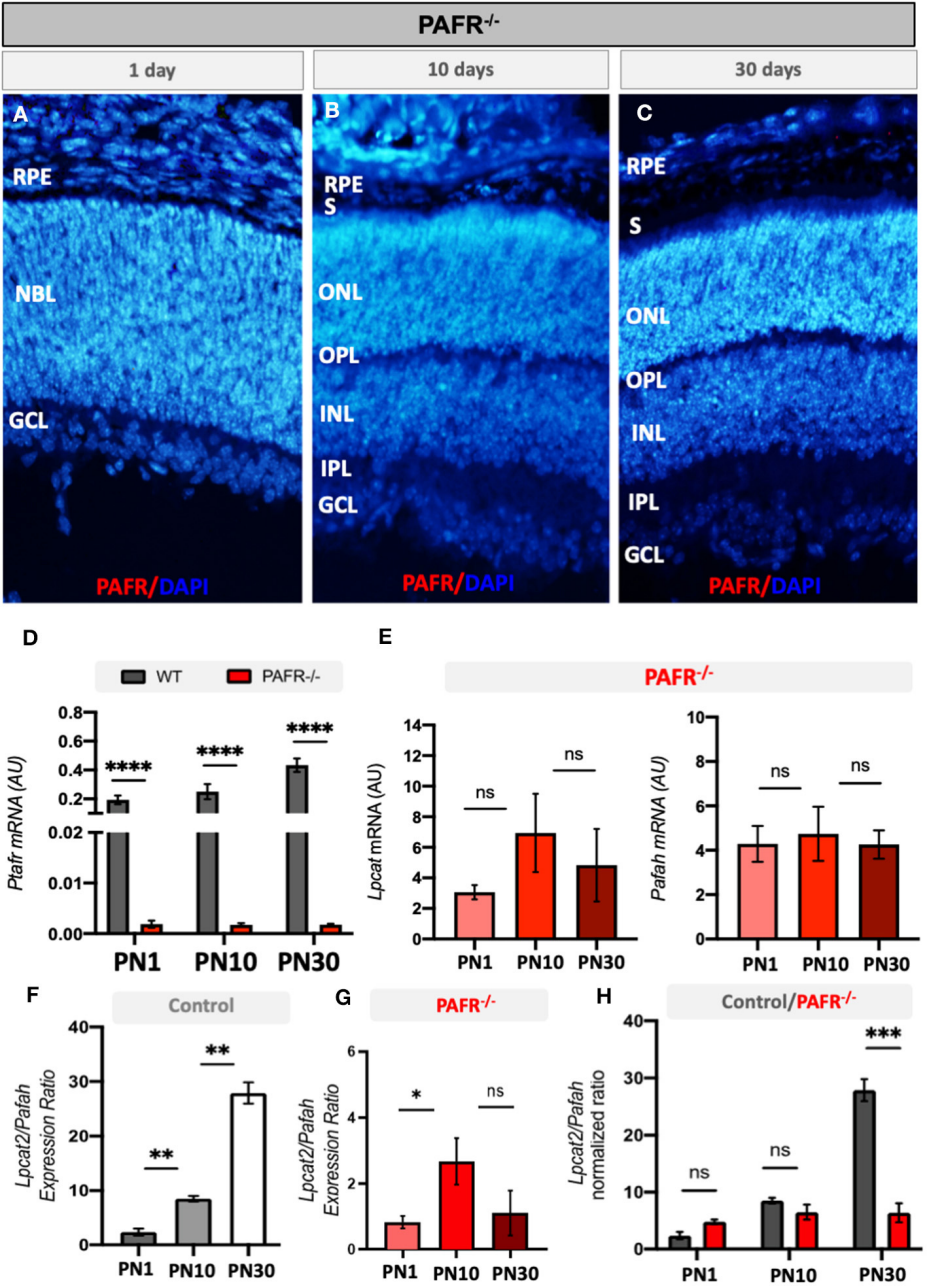
Analysis of PAFR knockout mice (PAFR). **(A–C)** Protein expression by immunohistochemistry of PAFR in PAFR ^−/−^ animals at postnatal day 1 (PN1), day 10 (PN10), and adult animals (PN30) (*n* = 4). **(D)** PAF receptor (*Ptafr*) transcriptional expression in the retinas of PN1, PN10, and PN30 wild-type (WT) animals and PAFR^−/−^ (*n* = 5). **(E)** *Lpcat* and *Pafah* mRNA expression in knockout animals at PN1, PN10, and PN30 (*n* = 5). *Lpcat* and *Pafah* ratio expression in **(F)** control animals and **(G)** PAFR^−/−^ animals (*n* = 5). **(H)** Comparative analysis on the *Lpcat* and *Pafah* ratio expression between WT and PAFR^−/−^ animals (*n* = 5). RPE, Retinal Pigmented Epithelium; NBL, neuroblastic layer; S, segments; ONL, outer nuclear layer; OPL, outer plexiform layer; INL, inner nuclear layer; IPL, inner plexiform layer; GCL, ganglion cell layer. Data are shown as mean ± S.E.M. ^*^P < 0.05; ^**^P < 0.01; ^****^P < 0.0001. ns, non-significant.

To assess the effect of PAFR deletion on retinal development and function, we analyzed the retinal molecular and electrophysiological profiles of PAFR knockout mice (PAFR^−/−^). First, PAFR ablation was confirmed by IHC and RT-PCR (Figure 3). Both PAFR protein (Figures 3A, C) and transcripts (Figure 3D) were not detected in the PAFR^−/−^ retinas at any of the time points studied. *Lpcat* and *Pafah* transcripts presented no significant changes between PN1, PN10, and PN30 animals (Figure 3E), suggesting that PAFR is absent in PAFR^−/−^ animals and PAF biogenesis enzymes are not regulated.

The ratio between PAF ligand production and degradation enzymes (Lpcat2/Pafah, respectively) indirectly indicates the levels of PAF molecules available in the system. Control animals gradually increased the ratio between *Lpcat* and *Pafah* transcriptional expression over time, indicating that *Lpcat* expression at PN10 overcomes the expression of *Pafah* in comparison to PN1 animals (Figure 3F). A similar correlation was observed at PN30 in comparison to PN10. This finding suggests that PAF synthesis by Lpcat is higher than degradation by Pafah during retinal differentiation in control animals.

Similar to control animals, PAFR^−/−^ animals presented a positive correlation of Lpcat/Pafah expression at PN10 in comparison to PN1 animals; however, no differences were detected in adult expression (Figure 3G), suggesting that PAF synthesis and degradation ratio become equilibrated in adults. Comparison of the Lpcat2/Pafah ratio between controls and PAFR^−/−^ animals indicated a significant difference in PN30 animals (Figure 3H), with a higher ratio in control animals.

### 3.3 PAFR ablation increases proliferation markers in RPC and decreases neural markers

**Figure 4:**
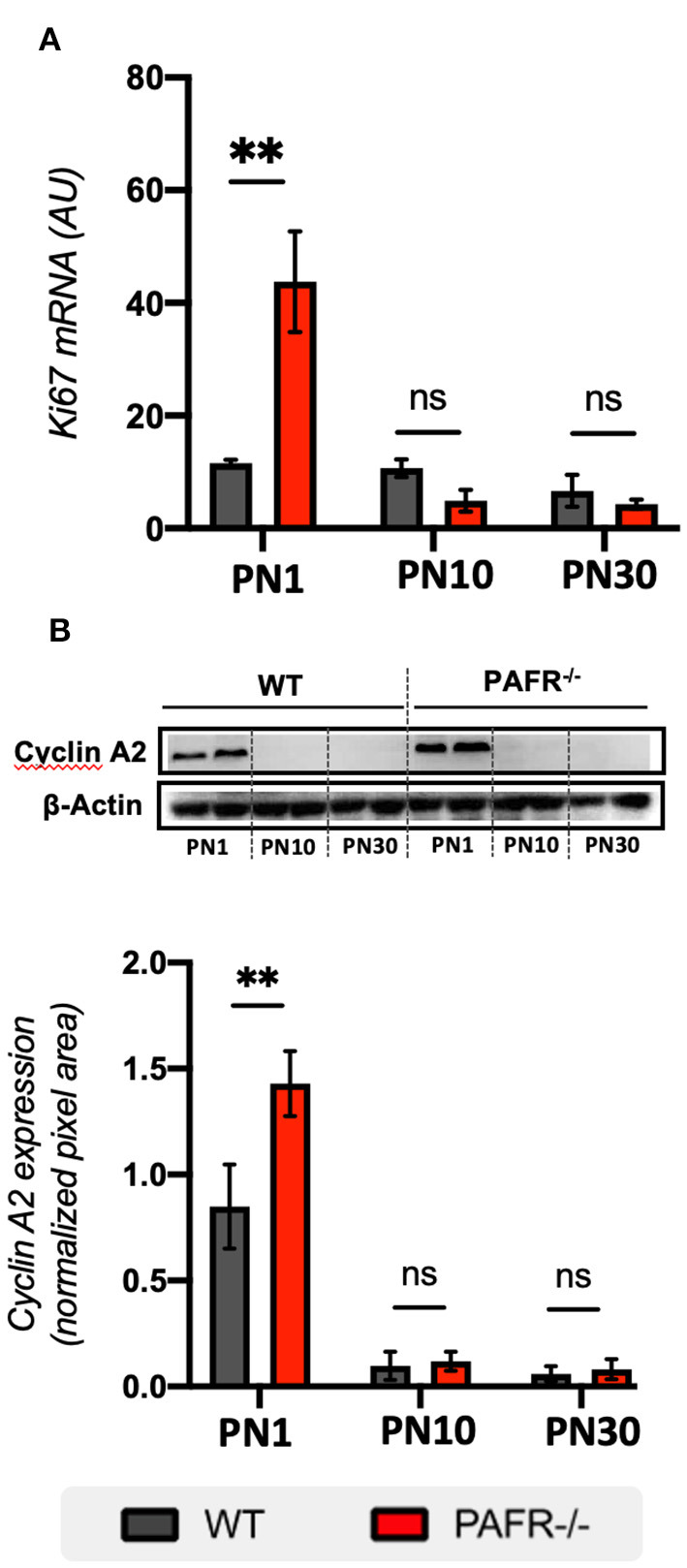
Cell cycle markers. **(A)** Transcriptional analysis on *Ki67* expression between wild-type (WT, gray) and PAFR^−/−^ animals (red) at postnatal day 1 (PN1), day 10 (PN10), and adult animals (PN30) (*n* = 5). **(B)** Protein analysis by Western blotting on *Cyclin A2* expression between wild type (WT, gray) and PAFR^−/−^ animals (red) at the same time points. Each band is representative of one independent sample, and four samples were analyzed per group (*n* = 4). Data are shown as mean ± S.E.M. ^**^P < 0.01. ns, non-significant.

To investigate cell proliferation status in PAFR^−/−^ animals, we first analyzed the transcriptional expression of Ki67, which is widely used to mark all cell cycle phases (S, G2, and M). Significant overexpression of *Ki67* was detected in PN1 PAFR^−/−^ animals compared to WT (Figure 4A). No differences were observed in *Ki67* levels at PN10 and PN30.

Cyclin A2 is known to be expressed in RPC at the late S and G2/M phases of PN0 mice retinas, with perinuclear and nuclear expression in cells near the apical surface of the NBL (Barton and Levine, 2008). At PN1, cyclin A2 was highly expressed in both PAFR^−/−^ and control animals, with significant overexpression in PAFR^−/−^ animals (Figure 4B). No differences in expression were detected at PN10 and PN30 in either group. Together, these results suggest that PAFR ablation increases the proliferation of RPC cells during the postnatal stage of late retinogenesis.

**Figure 5:**
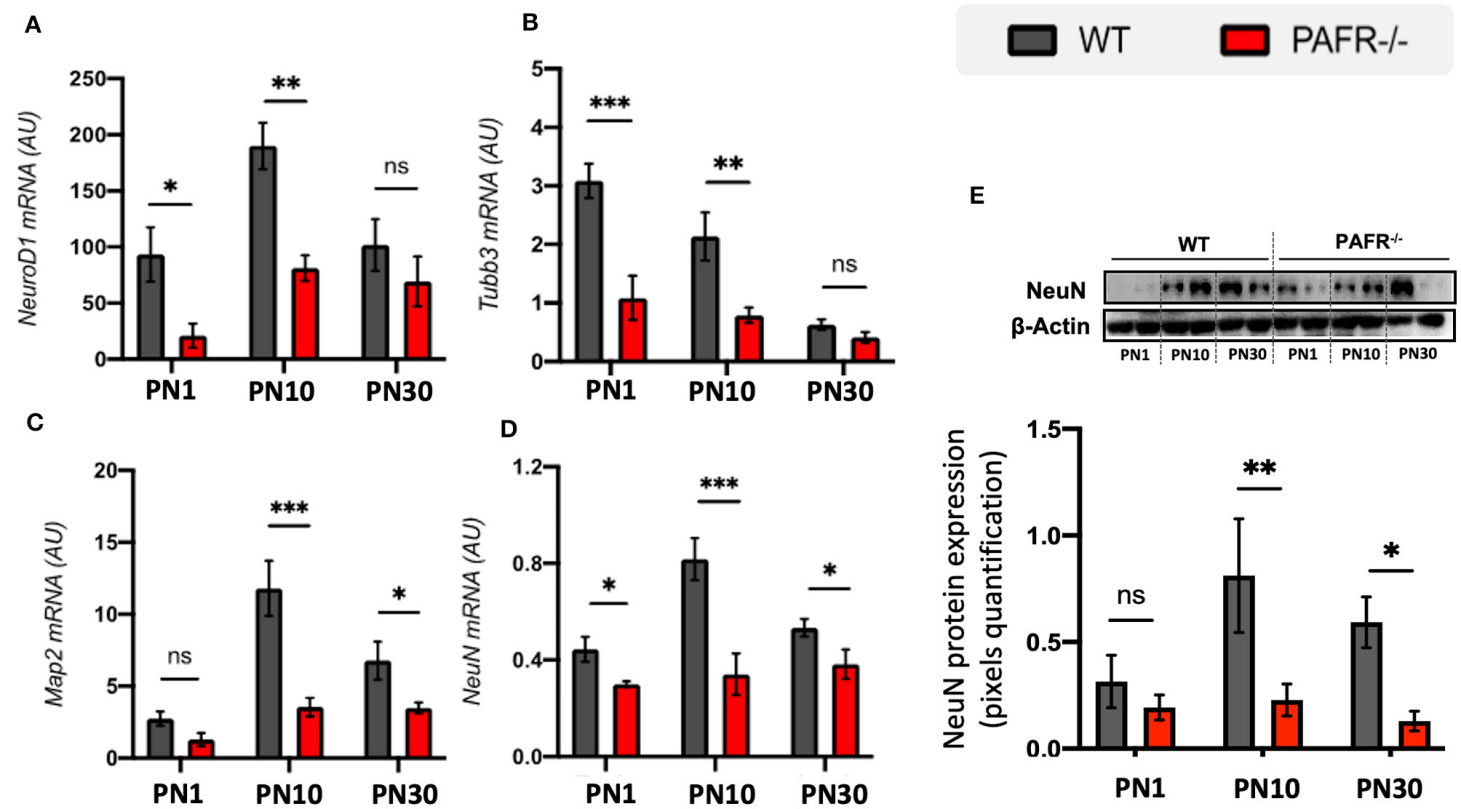
Pan-neuronal differentiation analysis of PAFR^−/−^ retinas. Transcriptional expression of early development neural markers: **(A)** Neuronal Development 1 (*NeuroD1*) and **(B)** β-Tubulin 3 (*Tubb3*) in wild-type (WT) animals (gray bars) in comparison to PAFR^−/−^ (red bars), at postnatal day 1 (PN1), day 10 (PN10), and adult animals (PN30) (*n* = 5). Similar transcriptional expression of matured neuronal markers: **(C)** microtubule-associated protein 2 (*Map2*), and **(D)** neuronal Nuclei (*NeuN*) (*n* = 5). **(E)** Western blotting analysis on NeuN protein levels at PN1, PN10, and PN30 WT and PAFR ^−/−^ animals. Each band is representative of one independent sample, and four samples were analyzed per group (*n* = 4). Data are shown as mean ± S.E.M. ^*^P < 0.05; ^**^P < 0.01; ^***^P < 0.001. ns, non-significant.

We next investigated the expression of the early neuronal markers NeuroD1 and class III β-tubulin (Tubb3). PAFR^−/−^ animals expressed significantly lower transcriptional levels of *NeuroD1* and *Tubb3* at PN1 and PN10 compared to controls but no differential expression at PN30 (Figures 5A, B). Map2 and NeuN are known to be expressed by differentiated neurons. PAFR knockout mice expressed lower levels of *Map2* at PN10 and PN30 compared to controls (Figure 5C), while *NeuN* transcripts were downregulated at all time points (Figure 5D). Western blotting analysis indicated lower NeuN protein expression at PN10 and PN30 in PAFR^−/−^ animals than controls (Figure 5E).

### 3.4 Selective retinal markers decreased in the absence of PAFR

**Figure 6:**
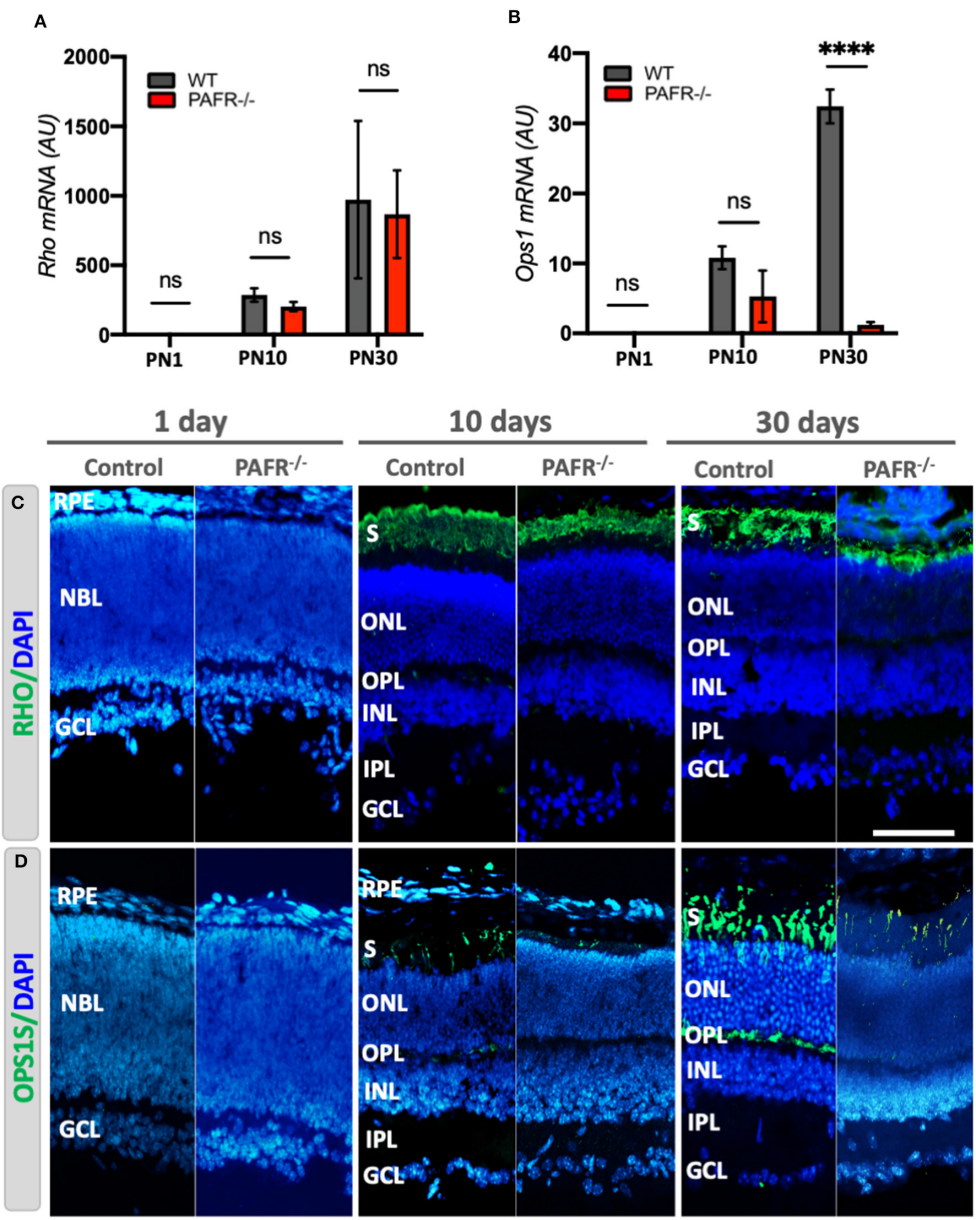
Photoreceptor analysis in PAFR^−/−^ mice retina. **(A)** mRNA expression of mature rod photoreceptors (Rhodopsin – *Rho*) in wild-type (WT) animals (gray bars) in comparison to PAFR^−/−^ (red bars) at postnatal day 1 (PN1), day 10 (PN10), and adult animals (PN30) (*n* = 5). **(B)** Transcriptional expression of differentiated cone photoreceptors (Opsin – *Ops1* = *OPNS1W*) at the same time points (*n* = 5). **(C)** Rhodopsin protein expression was not detected by immunohistochemistry at PN1 in either animal. In contrast, it was strongly detected at the photoreceptor segment layer and outer nuclear layer (ONL) at PN10 and PN30, with no differences between WT and PAFR^−/−^ retinas (*n* = 4). **(D)** Similar to rhodopsin, the opsin 1 expression was not detected at PN1 retinas but was detected at PN10 and PN30 more significantly in controls than in PAFR^−/−^ animals (*n* = 4). RPE, retinal pigmented epithelium; NBL, neuroblastic layer; S, segments; ONL, outer nuclear layer; OPL, outer plexiform layer; INL, inner nuclear layer; IPL, inner plexiform layer; GCL, ganglion cell layer. Data are shown as mean ± S.E.M. ^****^P < 0.0001. ns, non-significant. Scale bar = 100 μm.

We evaluated if the decrease in early neural marker expression detected in PAFR^−/−^ animals was reflected in the differentiation of retinal-specific cell types. We found that transcriptional expression of the mature rod marker rhodopsin (*Rho*) was similar in PAFR^−/−^ and WT animals at all stages (Figure 6A). However, the mature cone marker opsin (*Ops1*) was significantly downregulated in adult PAFR-null animals (Figure 6B). Since there was no information in the literature about photoreceptor marker expression in PAFR^−/−^ mice, we analyzed rhodopsin and opsin expression by immunohistochemistry and confocal microscopy. Our analysis detected no rhodopsin expression in PN1 retinas, with faint rhodopsin positive staining in the photoreceptor segment layer at PN10, becoming more prominent at PN30, and similar expression in both WT and PAFR^−/−^ animals (Figure 6C). Opsin expression, however, was notably reduced in PAFR^−/−^ at PN10 and PN30 (Figure 6D).

**Figure 7:**
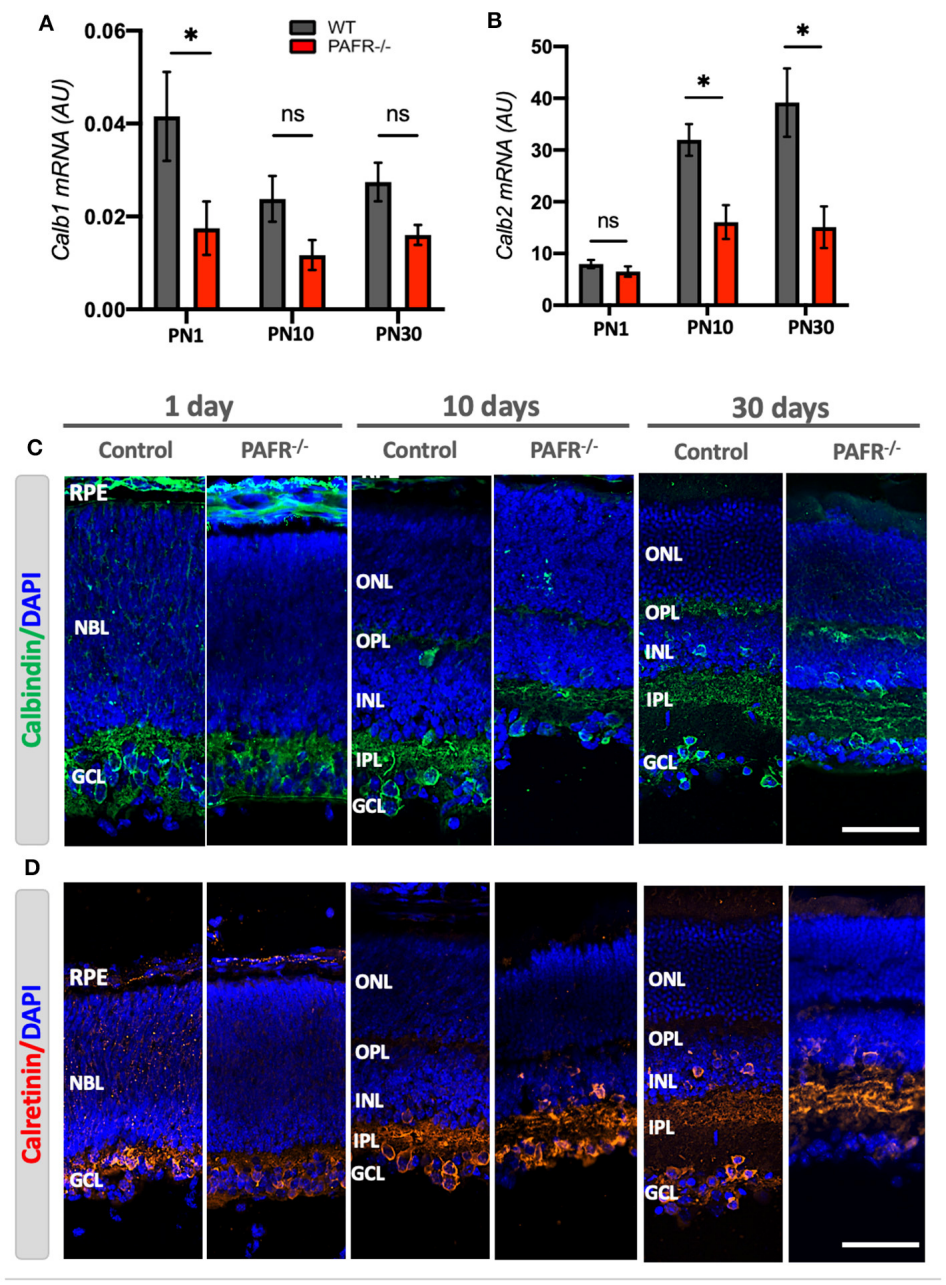
Retinal cell types differentiation analysis in PAFR^−/−^ mice retina. **(A)** mRNA expression of mature horizontal cells (Calbindin – *Calb1*) in wild-type (WT) animals (gray bars) in comparison to PAFR^−/−^ (red bars) at postnatal day 1 (PN1), day 10 (PN10), and adult animals (PN30) (*n* = 5). **(B)** Transcriptional expression of differentiated amacrine cells (Calretinin – *Calb2*) at the same time points (*n* = 5). **(C)** Calbindin protein (green) expression was detected by immunohistochemistry at RPE, NBL, and RGC at PN1 retinas from both wild-type (WT) and PAFR^−/−^ animals. At PN10, both animals presented calbindin at the plexiform layers, INL, and retinal ganglion cells within the RGC (*n* = 4). **(D)** Calretinin (red) was also detected at RPE, NBL, and GCL at PN1 retinas and INL, IPL, and GCL of both PN10 and PN30 retinas. Calretinin appears less expressed at the RGC of PN30 retinas from PAFR ^−/−^ animals than WT (*n* = 4). RPE, retinal pigmented epithelium; NBL, neuroblastic layer; S, segments; ONL, outer nuclear layer; OPL, outer plexiform layer; INL, inner nuclear layer; IPL, inner plexiform layer; GCL, ganglion cell layer. Data are shown as mean ± S.E.M. ^*^P < 0.05. ns, non-significant. Scale bar = 100 μm.

Next, we evaluated the Ca2^+^-binding buffer proteins calbindin (*Calb1*) and calretinin (*Calb2*) expression. Calbindin can be detected in fully differentiated horizontal cells and OPL, and calretinin in amacrines regularly located (somata in the INL) and displaced (somata in the GCL). Both markers are known to be expressed in a third of morphologically identified retinal ganglion cells and the dendritic stratification within the inner plexiform layer (IPL). Our data indicated that PAFR^−/−^ animals downregulated *Calb1* transcript expression at the early postnatal stage (Figure 7A) with no statistical differences compared to control animals at later stages. On the other hand, *Calb2* was downregulated in PAFR^−/−^ retina cells at PN10 and PN30 (Figure 7B). As expected, both markers were expressed in the newly formed RGC at PN1, the earlier layer developed during retinogenesis (Figures 7C, D). Both calbindin and calretinin were detected in the NBL of control animals with lower signals in PAFR^−/−^ mice retinas. Mean fluorescence intensity quantification analysis demonstrated lower expression of calbindin in PN1 retinas but no differences in calretinin expression between control and PAFR^−/−^ animals (Supplementary Figure 2A). At PN10 and 30, calbindin was observed at IPL, some horizontal cells at INL, and a few ganglion cells at the RGC layer with similar patterns and protein intensity expression in both animals (Figure 7C). Calretinin protein presented a similar expression pattern at RGC on PN1, and RGC, INL, and IPL at PN10 and PN30 for both animals (Figure 7D). Observations indicated fewer positive cells in the RGC layer at PN30 PAFR^−/−^ animals than controls, based on the lower mean fluorescence expression detected in the animals' retinas (Supplementary Figure 2B).

No differences in transcriptional expression of Müller glial differentiated markers were observed in PAFR^−/−^ at any time points investigated (data not shown). Together, these results suggest that the absence of PAFR could preferentially affect the maturation of the cells born during early retinogenesis (such as cones, amacrines, and horizontal cells).

### 3.5 PAFR^−/−^ mice have altered synapsis markers expression and electrophysiological responses

**Figure 8:**
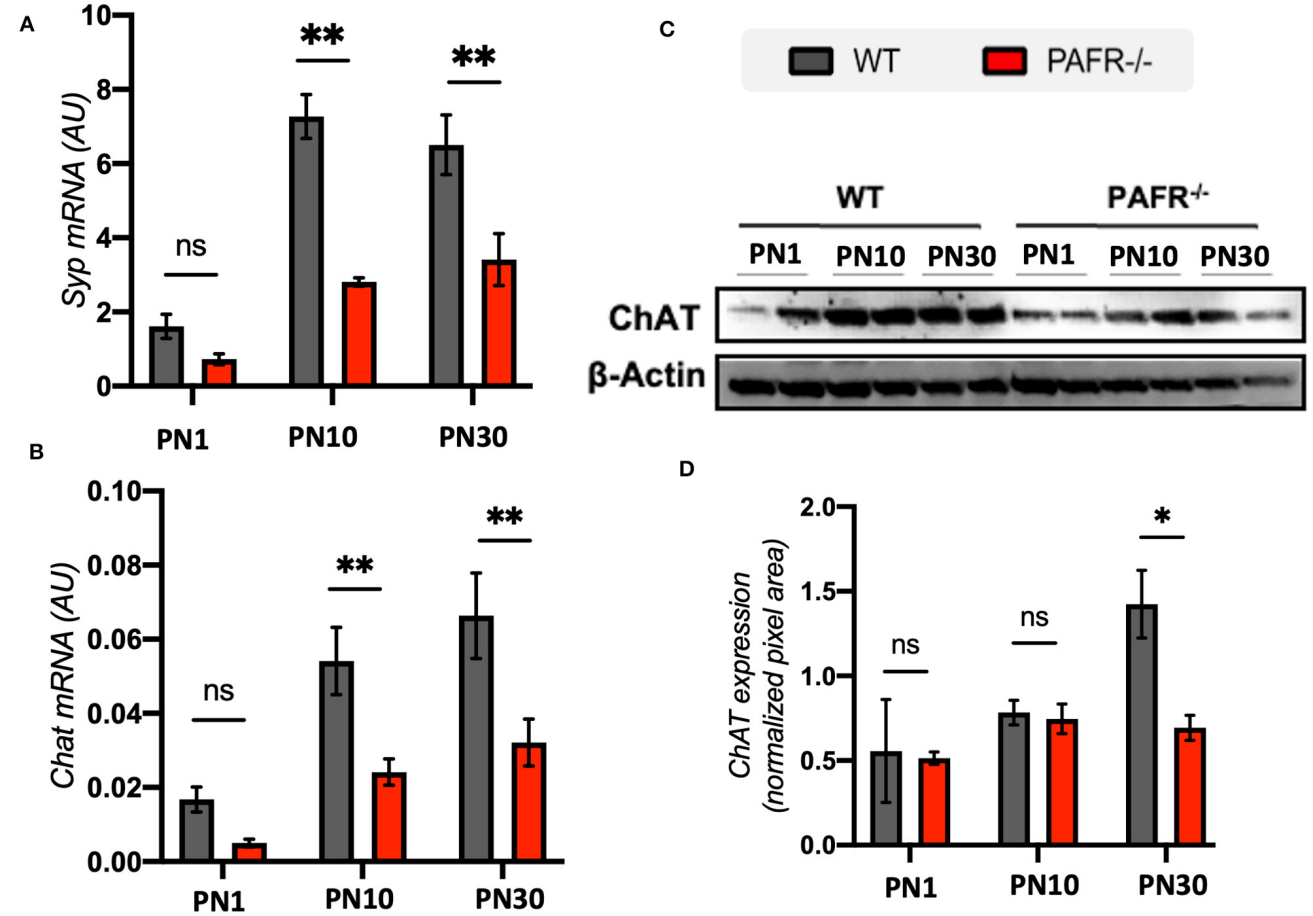
Synapse markers in PAFR^−/−^ mice retina. **(A)** Transcriptional expression of the synaptic maturation marker synaptophysin (*Syp*) in wild type (WT) animals (gray bars) in comparison to PAFR^−/−^ (red bars), at postnatal day 1 (PN1), day 10 (PN10), and adult animals (PN30) (*n* = 5). **(B)** Similar transcriptional analysis for choline acetyl-transferase (*ChAT*) (*n* = 5). **(C)** Western blotting analysis and **(D)** quantification of ChAT protein levels at PN1, PN10, and PN30 WT and PAFR ^−/−^ animals. Each band is representative of one independent sample, and four samples were analyzed per group (*n* = 4). Data are shown as mean ± S.E.M. ^*^P < 0.05; ^**^P < 0.01. ns, non-significant.

Presynaptic protein synaptophysin (*Syp*) transcriptional expression indicated lower levels at PN1 in both control and PAFR^−/−^ animals (Figure 8A). This result was expected once synaptophysin was first detected in the IPL and OPL of the mouse retina shortly before eye opening (around PN12). An important difference in *Syp* expression was observed at PN10 and PN30 time points, with significantly lower levels detected in PAFR^−/−^ animals than controls. Choline acetyltransferase (ChAT) presented a similar differential expression, with downregulated levels at PN10 and PN30 in PAFR-null animals (Figure 8B). Lower ChAT protein expression in PAFR^−/−^ retinas was detected in PN30 PAFR^−/−^ animals (Figures 8C, D).

**Figure 9:**
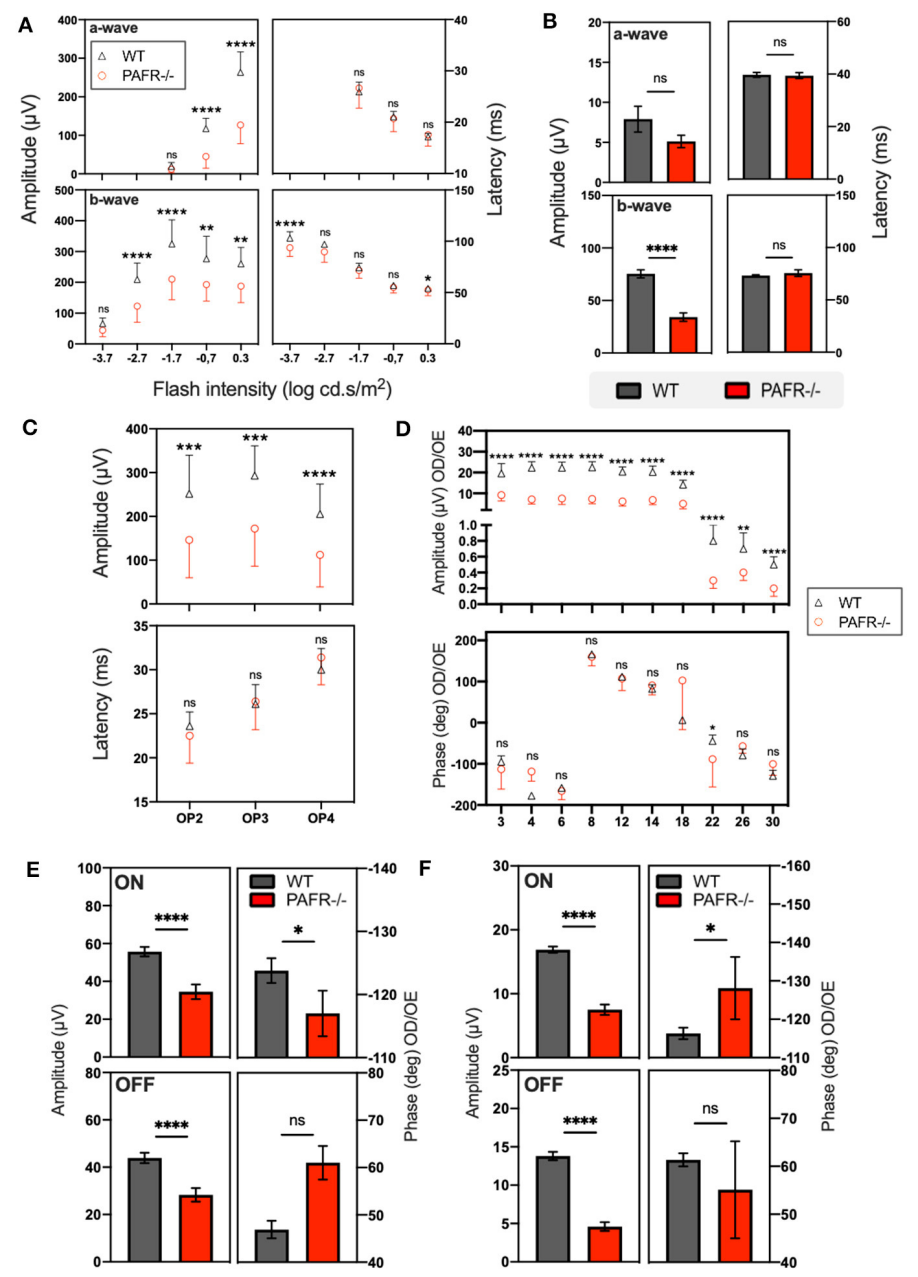
Electroretinogram (ERG) analysis. **(A)** Scotopic analysis on a-wave and b-wave amplitudes from wild-type (WT) animals (gray lines and bars) in comparison to PAFR^−/−^ (red lines and bars) at postnatal day 30 with 5 different intensities (*n* = 10). **(B)** Photopic analysis on a-wave and b-wave amplitudes from WT animals compared to PAFR^−/−^ (*n* = 10). **(C)** Oscillatory potentials (OP) analysis. **(D)** Sine wave stimuli (flicker) at different temporal frequencies: 3, 4, 6, 8, 12, 14, 18, 22, 26, and 30 Hz (*n* = 10). **(E)** Sawtooth stimuli with rapid-on increment and rapid-off decrement recorded in mesopic and photopic conditions at 4 Hz (*n* = 10). **(F)** Photopic ON/OFF responses (*n* = 10). Data are shown as mean ± S.E.M. ^*^P < 0.05; ^***^P < 0.001; ^****^P < 0.0001. ns, non-significant.

Finally, to check if the differences observed in retinal markers and synapsis could reflect on retinal function, we performed electroretinography (ERG) in adult PAFR^−/−^ and WT mice (Figure 9). Briefly, the ERG assesses the overall electrical responses of the retina, representing the coordinated synaptic activity of photoreceptors, bipolar cells, horizontal cells, and ganglion cells in response to visual stimuli. These stimuli can include light flashes or specific visual patterns with varying intensities and frequencies, which can stimulate the retina, resulting in a biphasic electrical wave composed of negative (a-wave) and positive (b-wave) components. The a-wave reflects the photoreceptor's membrane hyperpolarization, and the b-wave reflects the photoreceptor's postsynaptic activity in the inner nuclear layer (ON bipolar cells). Therefore, ERG can evaluate retinal electric response efficiency, analyze specific retinal neurons' synaptic competence, and monitor the different stages of visual processing. The data of means and standard deviation (SD) of ERG analysis are listed in Supplementary Table 1.

First, scotopic ERG revealed that PAFR^−/−^ mice have decreased a-wave amplitude at −0.7 (*p* < 0.001) and 0.3 (*p* < 0.001) but not at −1.7 log cd.s/m^2^, suggesting that PAFR^−/−^ adult retinas have altered photoreceptors' synaptic functions. Any differences were observed for the implicit times of a-wave among groups (Figure 9A). The scotopic b-wave amplitude was also decreased at −2.7 (*p* < 0.001), −1.7 (*p* < 0.001), −0.7 (*P* = 0.002), and 0.3 (*p* = 0.001) but not at −3.7 log cd.s/m^2^. There is also a decrease in the implicit times of b-wave at −3.7 (*p* < 0.001) and 0.3 (*p* < 0.05) but not at −2.7, −1.7, and −0.7 log cd.s/m^2^ (Figure 9A), suggesting post-receptor changes with on-bipolar cell impairment. The oscillatory potentials (OP) analysis revealed a decrease in the amplitude of PAFR^−/−^ mice for OP2 (*p* < 0.01), OP3 (*p* = 0.001), and OP4 (*p* < 0.001), with no difference for the OPs implicit times, suggesting an impairment at the signal processing of interneurons (amacrine cells) (Figure 9C).

The photopic ERG analysis showed that PAFR^−/−^ mice have decreased b-wave amplitude (*p* < 0.001) with no change in the implicit time, suggesting bipolar cells' abnormal function (Figure 9B).

To evaluate the integrity of the post-receptor mechanisms of the cone system, which act in the processing of temporal luminance, sine wave stimuli (flicker) were used at different temporal frequencies: 3, 4, 6, 8, 12, 14, 18, 22, 26, and 30 Hz. The amplitude and phase of the first harmonic were analyzed. We observed a decrease in the amplitude of PAFR^−/−^ retinas among all evaluated frequencies (3 Hz *p* < 0.001, 4 Hz *p* < 0.001, 6 Hz *p* < 0.001, 8 Hz *p* < 0.001, 12 Hz *p* < 0.001, 14 Hz *p* < 0.001, 18 Hz *p* < 0.001, 22 Hz *p* < 0.001, 26 Hz *p* < 0.01, 30 Hz *p* < 0.001). For the phase, we only observed a decrease at 22 Hz (*p* < 0.05), suggesting an alteration in the cone-driven system (Figure 9D).

Finally, Sawtooth stimuli with rapid-on increment and rapid-off decrement were recorded in mesopic and photopic conditions at 4 Hz, allowing us to evaluate the integrity of ON/OFF visual pathways. The analysis of mesopic responses showed a decrease for the ON (*p* < 0.001) and OFF (*p* < 0.001) amplitudes of the first harmonic on PAFR^−/−^ mice, with an increase for the ON phase (*p* < 0.05), suggesting a delayed synaptic response. No changes were found for the OFF phase in mesopic conditions (Figure 9E). The analyses of the photopic ON/OFF responses, as for the mesopic condition, showed a decrease of ON (*p* < 0.001) and OFF (*p* < 0.001) amplitudes of the first harmonic, with a decrease for the ON phase (*p* < 0.05) on PAFR^−/−^ mice compared to the wild type (Figure 9F), strongly indicating altered cones synaptic responses. Together, these data suggest that PAFR ablation could decrease synaptic machinery expression and synaptic responses, reflecting alterations of ON/OFF pathways.

## 4 Discussion

Although it is known that PAF suppresses RPC proliferation during retinal development, its effects on retinal cell differentiation dynamics were not explored. Here, we provide evidence that PAF-related enzymes and PAFR expression are regulated during mammalian retinal development and differentiation.

First, we showed that transcripts of PAFR, LPCAT2, and PAFAH presented similar expression patterns in both human and mouse retinas, suggesting that PAF has a conservative mechanism. In both species, the PAF receptor and its synthesis enzyme increased during retinal maturation, while the degradation enzyme decreased in adult retinas. The correlations between PAFR, PAF synthesis, and degradation enzymes have been extensively reported in neuronal development and neurobiological functions (Hattori and Arai, 2015). Similar to our results, the mouse postnatal brain showed LPCAT activity at critical neuronal maturation points, with a progressive increase starting at PN10 and settling during adulthood (Eto et al., 2020). The regulatory PAF-AH β-subunit, the LIS1 protein, was shown to be an essential regulator of neural progenitors' cell proliferation and migration (Jheng et al., 2018; Rolland et al., 2021; Penisson et al., 2022) since the LIS1 mutation causes severe brain development impairment and was determined to be the main cause of type 1 lissencephaly (Hines et al., 2018).

In conjunction with the data in the literature, our results strongly indicate that the PAF pathway is present during retinal maturation. However, information about the precise starting date of PAF-, PAFR-, and PAF-related enzymes expression during retinal embryonic development, as well as the cell types that are the source of PAF production and secretion for the neighboring cells in the retina, is still poorly known at the moment.

Fragel-Madeira et al. (2011) observed that Müller cells act as an important source of PAF in the developing retina. We detected the transcriptional expression of the PAF regulatory network as early as 52–57 gestational days in humans and PN1 in mice. These time points mark the development of cell types born before the Müller glial cells, suggesting that PAF could also be expressed by other cell types.

Locally produced PAF is postulated to exert its influence over short distances, traversing the plasma membrane of the producing cell and reaching adjacent cells. This may occur by binding to PAFR on the plasma membrane or traversing the membrane to engage the nuclear receptor. The biochemical nature of PAF, characterized as a bioactive lipid, can induce pathological or physiological activities in picomolar orders. Actions over long distances, as observed in typical endocrine signaling, are not commonly ascribed to this molecule. This information strongly suggests that different cell types produce PAF in the retina during its development and that the release of this molecule directly influences the surrounding cells.

It is also known that PAFR and PAF-related enzymes can exert direct effects on neuroblast cell proliferation. For instance, the catalytic PAF-AH α-subunits were shown to modulate the Wnt pathway, an important regulator of neural progenitor cell proliferation and neuronal lineage determination (Livnat et al., 2010). In addition, exogenous stimulation of PAFR arrested RPC's cell cycle at the S/G2 phase transition, decreasing neuroblast nuclear migration and downregulating cyclin B1 expression (Fragel-Madeira et al., 2011).

Our results demonstrated the influence of PAFR and PAF-related enzymes regulation on the expression of two important cell cycle markers, Ki67 and Cyclin A2. We observed that PAFR^−/−^ mice significantly increased the expression of both markers during postnatal retinal development.

Once the absence of PAF signaling increased proliferation, it was expected to have a cascade effect on retinal neuronal differentiation and maturation. Initially, we detected a decrease in early neural transcripts (*NeuroD1* and *Tubb3*) at PAFR^−/−^ PN1 and PN10 retinas. This is reflected in the lower expression of the mature neural markers *NeuN* and *Map2* at later stages of differentiation, such as PN10 and PN30. Finally, this disruption affected the expression of specific markers of mature cells, such as cone photoreceptors (*Ops*), amacrine, and horizontal cells (calbindin and calretinin). Interestingly, the affected cells are known to be born at an early stage of retinogenesis. Fully differentiated rod photoreceptors, born mostly at late retinogenesis, apparently were not significantly affected.

Accumulated pieces of evidence have proposed PAF/PAFR signaling as a potent modulator of CNS processes, particularly those related to neuronal plasticity and neuroprotection in both non-pathological and inflammatory or neurodegenerative conditions (Liu et al., 2017; Zhao et al., 2021; Luo et al., 2022). Neuroblasts stimulated with PAF arrested cell proliferation, increased neuronal differentiation phenotype, and upregulated synaptic responses, enhancing intracellular calcium and ATP release (Kornecki and Ehrlich, 1988). In this study, the PAF effect on neuronal differentiation was completely inhibited by PAFR antagonist (CV-3988) treatment, suggesting that the effects of PAF are important for neural differentiation and maintenance.

The endpoint of the neural cell differentiation process is to generate a fully competent and responsive neuron capable of synapsis transmission and physiological response to stimuli. PAFR^−/−^ mice retina have reduced the expression of synaptogenesis markers such as choline acetyl-transferase (*ChAT*) and synaptophysin (*Syp*). Syntaxin expression presented inconclusive results (data not shown). The modulation of ChAT and Syp could be the cause of the decreased a-wave and diminished b-wave amplitudes and implicit time observed in PAFR^−/−^ animals compared to control wild-type (WT). These results indicated abnormal photoreceptors and bipolar cells' physiological activities, as well as impaired neuronal synaptic transmissions, suggesting the presence of dysfunctional neuronal activity and/or unappropriated neuronal synapses in PAFR^−/−^ retinas.

There has been little investigation into the downstream signaling pathways or molecular events within the synaptic bouton mediated by PAF signaling. Previous studies suggested that PAFR may modulate synaptic pattern responses and neurotransmitter release in various CNS tissues (Kornecki and Ehrlich, 1988). Hammond et al. (2015) demonstrated that PAF increased presynaptic vesicle exocytosis through PKC activation and elevated intracellular calcium within presynaptic boutons. They also indicated increased phosphorylation of synapsin I and greater dispersion of synapsin I from synaptic vesicles when primary hippocampal cultures were exposed to PAF.

Syntaxins are membrane proteins localized to the presynaptic plasma membrane and are involved in vesicle fusion. They mediate the fusion of synaptic vesicles into the plasma membrane. We investigated syntaxin expression; however, the results were inconclusive. We believe that these inconclusive results are because PAF is a lipid mediator, and syntaxins are located in the synaptic vesicle (consisting primarily of phospholipids). Due to its importance in synapsis dynamics, syntaxin expression could be highly modulated in these vesicles, and compensatory mechanisms could interfere with its regulation.

PAF/PAFR are known to act as second messengers in CNS-specific excitatory synapses, increasing the amplitude of postsynaptic currents and decreasing the latency of presynaptic action potentials (Clark et al., 1992; Marcheselli and Bazan, 1994; Chen et al., 2001). PAFR^−/−^ animals decreased neuronal synaptic activity and long-term potentiation in different brain regions, such as the hippocampus (Ishii et al., 1998). It is believed that PAFR-mediated synaptic modulation involves intracellular calcium modulation, kinase activities, and synaptic vesicle exocytosis (Doly et al., 1987; Bussolino et al., 1988, 1989; Moriguchi et al., 2010; Hammond et al., 2015).

In this study, we demonstrated that the abnormal differentiation and physiological effects observed in fully differentiated retinal neurons result from a chain of events that begins during retinogenesis. During the neurogenic program, appropriate cell cycle entry and exit regulation are essential to control cell fate determination (Miles and Tropepe, 2016). For example, cell cycle phase lengthening is widely associated with precursor cells undergoing neural cell fate (Hardwick and Philpott, 2014). Inhibition of cyclin-dependent kinases induced premature neurogenesis, while CyclinD1 overexpression in neural stem cells delayed neurogenesis, decreasing the differentiation process of late-born neurons (Calegari and Huttner, 2003; Lange et al., 2009). We believe that the altered balance of proliferation observed in PAFR^−/−^ could ignite the neuronal changes observed at later points. To confirm this hypothesis, more investigation is necessary.

Our results also suggested that the effects of PAF ablation were observed in cones, amacrines, and horizontal cells, known to be born during the early neurogenesis that occurs around embryonic days 10–12 in mice. It means that the PAFR and PAF regulatory mechanisms could be present as early as the determination of the eye field gene expression in the neural tube. To confirm this study, experiments conducted during the early embryonic stages or retinal organoid cultures would be necessary.

## Data availability statement

The datasets presented in this study can be found in online repositories. The names of the repository/repositories and accession number(s) can be found in the article/Supplementary material.

## Ethics statement

Ethical approval was not required for the study involving humans in accordance with the local legislation and institutional requirements. Written informed consent to participate in this study was not required from the participants or the participants' legal guardians/next of kin in accordance with the national legislation and the institutional requirements. The animal study was approved by Ethical Committee for Animal Research of the Institute of Biomedical Sciences of the University of Sao Paulo (protocol number #3588090419). The study was conducted in accordance with the local legislation and institutional requirements.

## Author contributions

BD: Conceptualization, Formal analysis, Investigation, Methodology, Writing – original draft, Writing – review & editing. AL: Investigation, Methodology, Writing – review & editing. DV: Conceptualization, Writing – review & editing. SJ: Conceptualization, Writing – review & editing. CD: Conceptualization, Formal analysis, Funding acquisition, Methodology, Supervision, Writing – original draft, Writing – review & editing.

## Supplementary material

Supplementary Figure 1 Detailed PAFR protein expression. **(A–C)** Postnatal day 1 (PN1) retinas expressed PAFR in the retinal pigmented epithelium (RPE) at the outermost region of the neuroblastic layer (future photoreceptor layer) and the newly formed retinal ganglion cell (RGC) layer. **(D–F)** At PN10, PAFR was detected in RPE and strongly expressed in the photoreceptor segment layer (S), in the outer and inner plexiform layers (OPL and IPL), in the inner nuclear layer (INL), and at the RGC layer. **(G–I)** Adult retinas at PN30 expressed PAFR mainly at the photoreceptor segments, plexiform layers, INL, and RGC. Nuclear staining with DAPI (blue), PAFR (red), and merged image in **(C, F, I)**. *N* = 4.

Supplementary Figure 2 Calbindin and calretinin protein expression. Mean fluorescence intensity of **(A)** Calbindin and **(B)** Calretinin, measured after confocal analysis of wild-type (WT) animals (gray bars) in comparison to PAFR^−/−^ (red bars), at postnatal day 1 (PN1), day 10 (PN10), and adult animals (PN30). Data are shown as mean ± S.E.M. ^*^*P* < 0.05. ns, non-significant. *N* = 4.
