# Supplementary figures and images for: Platelet-activating factor receptor (PAFR) regulates neuronal maturation and synaptic transmission during postnatal retinal development
Source: Front Cell Neurosci. 2024 Mar 20;18:1343745. doi: 10.3389/fncel.2024.1343745 (PMC10988781; doi:10.3389/fncel.2024.1343745)

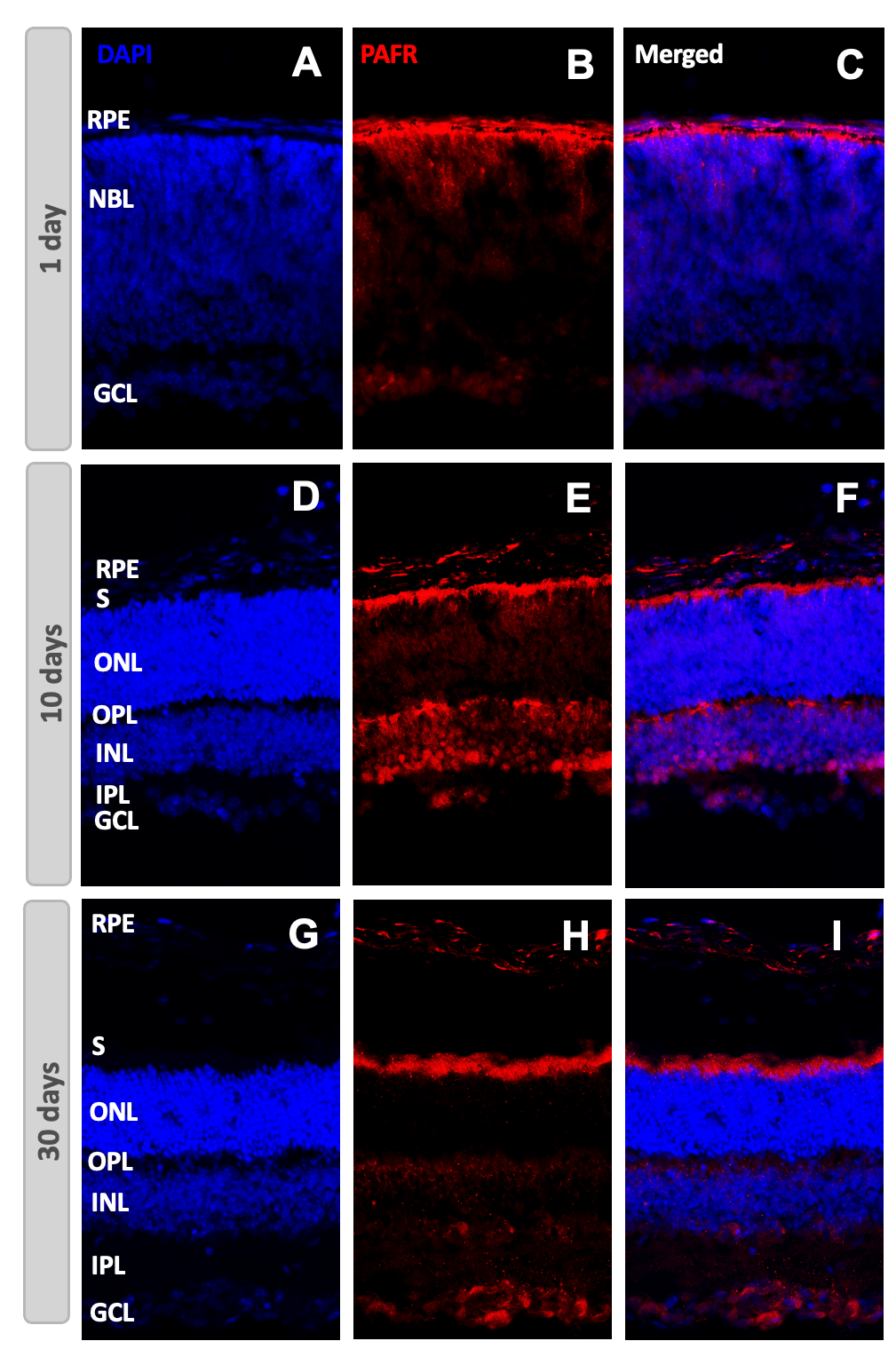

Supplement: Supplementary Figure 1 — Detailed PAFR protein expression. (A–C) Postnatal day 1 (PN1) retinas expressed PAFR in the retinal pigmented epithelium (RPE) at the outermost region of the neuroblastic layer (future photoreceptor layer) and the newly formed retinal ganglion cell (RGC) layer. (D–F) At PN10, PAFR was detected in RPE and strongly expressed in the photoreceptor segment layer (S), in the outer and inner plexiform layers (OPL and IPL), in the inner nuclear layer (INL), and at the RGC layer. (G–I) Adult retinas at PN30 expressed PAFR mainly at the photoreceptor segments, plexiform layers, INL, and RGC. Nuclear staining with DAPI (blue), PAFR (red), and merged image in (C, F, I). N = 4. [file Image_1.TIF]

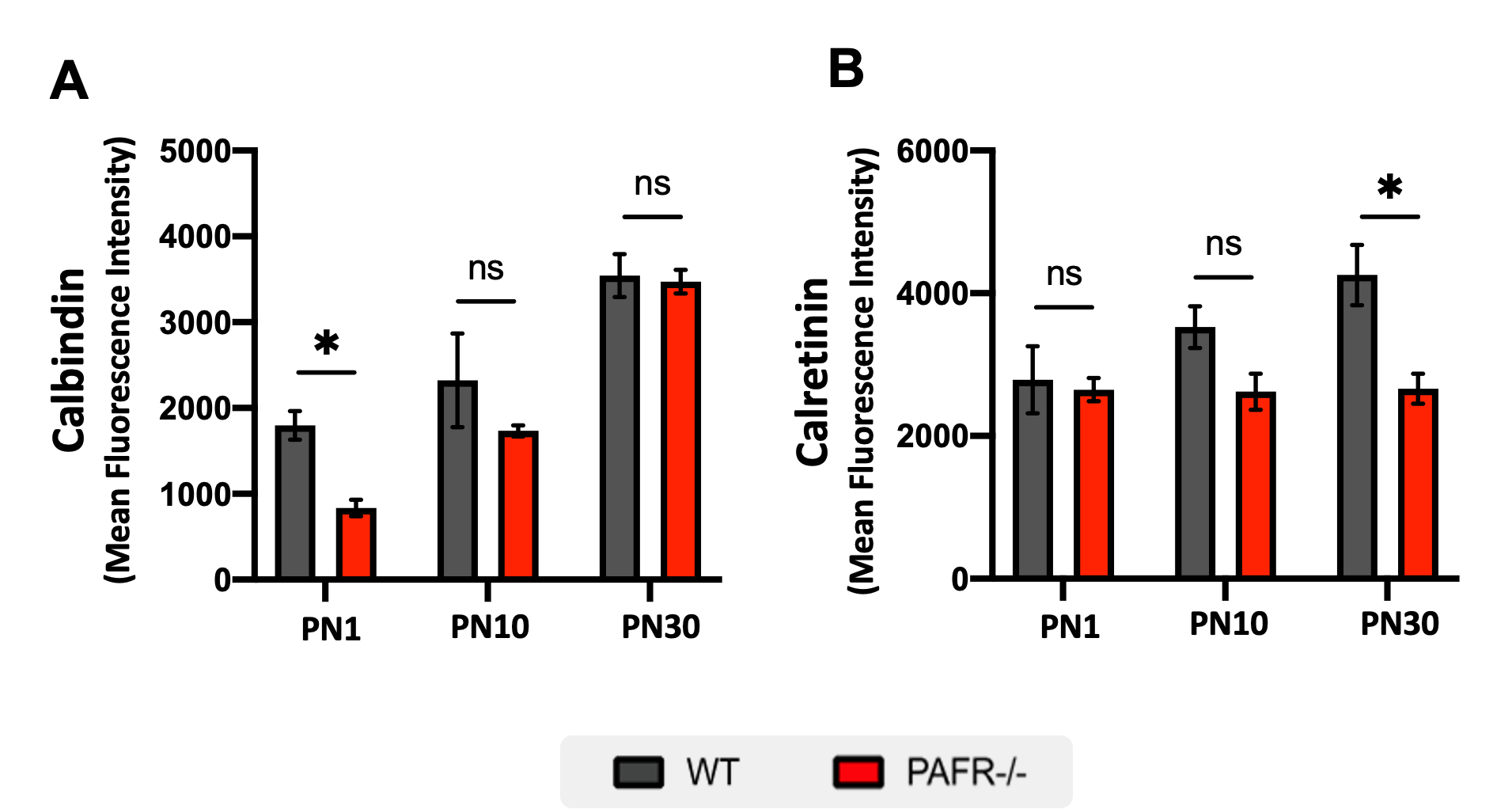

Supplement: Supplementary Figure 2 — Calbindin and calretinin protein expression. Mean fluorescence intensity of (A) Calbindin and (B) Calretinin, measured after confocal analysis of wild-type (WT) animals (gray bars) in comparison to PAFR−/− (red bars), at postnatal day 1 (PN1), day 10 (PN10), and adult animals (PN30). Data are shown as mean ± S.E.M. *P < 0.05. ns, non-significant. N = 4. [file Image_2.TIF]
