# Supplementary material for: Platelet-activating factor receptor (PAFR) regulates neuronal maturation and synaptic transmission during postnatal retinal development
Source: Front Cell Neurosci. 2024 Mar 20;18:1343745. doi: 10.3389/fncel.2024.1343745 (PMC10988781; doi:10.3389/fncel.2024.1343745)
Supplement: Supplementary file 3 [file Table_1.pdf]

Supplementary Table 1. ERG means and standard deviation values.

Scotopic ERG		
**a-wave**	**Amplitude**	**Latency**
**-1.7**	WT = 19.3 ± 10.1; PAFR$^{-/-}$ = 12.0 ± 8.7	WT = 25.9 ± 1.8; PAFR$^{-/-}$ = 26.6 ± 3.9
**-0.7**	WT = 117.6 ± 26.5; PAFR$^{-/-}$ = 45.2 ± 30.7; P = 0.000000	WT = 21.0 ± 1.0; PAFR$^{-/-}$ = 20.7 ± 2.5
**0.3**	WT = 263.4 ± 53.0; PAFR$^{-/-}$ = 126.8 ± 48.3; P = 0.000000	WT = 17.1 ± 0.6; PAFR$^{-/-}$ = 17.5 ± 2.1
**b-wave**	**Amplitude**	**Latency**
**-3.7**	WT = 103.0 ± 6.3; PAFR$^{-/-}$ = 93.7 ± 8.5	WT = 103.0 ± 6.2; PAFR-/- = 93.7 ± 8.5; P = 0.0009
**-2.7**	WT = 209.3 ± 53.2; PAFR$^{-/-}$ = 122.6 ± 52.3; P = 0.00002	WT = 74.1 ± 4.4; PAFR-/- = 71.5 ± 7.5
**-1.7**	WT = 324.6 ± 77.9; PAFR$^{-/-}$ = 210.1 ± 66.7; P = 0.0004	WT = 97.1 ± 2.3; PAFR-/- = 89.6 ± 10.1
**-0.7**	WT = 276.4 ± 72.9; PAFR$^{-/-}$ = 192.6 ± 53.3; P = 0.002	WT = 56.9 ± 2.7; PAFR-/- = 55.0 ± 5.4
**0.3**	WT = 260.1 ± 53.0; PAFR$^{-/-}$ = 187.7 ± 53.2; P = 0.001	WT = 54.0 ± 2.2; PAFR-/- = 52.5 ± 5.5; P = 0.03

Photopic ERG		
	**Amplitude**	**Latency**
**a-wave**	WT = 7.9 ± 5.6; PAFR$^{-/-}$ = 5.1 ± 2.7	WT = 39.7 ± 3.2; PAFR$^{-/-}$ = 39.4 ± 3.9
**b-wave**	WT = 75.4 ± 13.3 PAFR$^{-/-}$ = 34.2 ± 14.0; P = 0.000000	WT = 73.2 ± 2.6 PAFR$^{-/-}$ = 75.5 ± 11.0

Oscillatory Potentials (OP) ERG		
	**Amplitude**	**Latency**
**OP2-time**	WT = 251.9 ± 87.8; PAFR$^{-/-}$ = 146.2 ± 86.2; P = 0.007424	WT = 23.6 ± 1.6; PAFR$^{-/-}$ = 22.5 ± 3.1
**OP3-time**	WT = 293.1 ± 68.2; PAFR$^{-/-}$ = 172.3 ± 86.2; P = 0.001089	WT = 26.1 ± 2.2 PAFR$^{-/-}$ = 26.4 ± 3.2
**OP4-time**	WT = 205.5 ± 68.2; PAFR$^{-/-}$ = 112.2 ± 73.3; P = 0.000936	WT = 30.0 ± 2.4 PAFR$^{-/-}$ = 31.4 ± 3.1

Flicker ERG		
	**Amplitude**	**Phase**
**3 Hz**	WT = 19.6 ± 4.7; PAFR$^{-/-}$ = 9.2 ± 2.9; P = 0.000151	WT = -94.8 ± 14.2; PAFR$^{-/-}$ = -113.2 ± 48.1
**4 Hz**	WT = 22.3 ± 2.9; PAFR$^{-/-}$ = 7.1 ± 2.2; P = 0.000001	WT = -177.5 ± 3.2; PAFR$^{-/-}$ = -118.8 ± 23.4
**6 Hz**	WT = 22.3 ± 2.7; PAFR$^{-/-}$ = 7.5 ± 2.8; P = 0.000000	WT = -158.2 ± 4.7; PAFR$^{-/-}$ = -165.8 ± 21.0
**8 Hz**	WT = 22.5 ± 2.7; PAFR$^{-/-}$ = 7.3 ± 2.3; P = 0.000000	WT = 166.2. ± 5.3; PAFR$^{-/-}$ = 160.1 ± 21.9
**12 Hz**	WT = 20.4 ± 2.3; PAFR$^{-/-}$ = 6.1 ± 2.2; P = 0.000000	WT = 111.5 ± 5.6; PAFR$^{-/-}$ = 107.3 ± 29.4
**14 Hz**	WT = 20.3 ± 2.8; PAFR$^{-/-}$ = 6.8 ± 2.2; P = 0.000000	WT = 82.4 ± 9.5; PAFR$^{-/-}$ = 90.8 ± 23.3
**18 Hz**	WT = 14.2 ± 2.1; PAFR$^{-/-}$ = 5.0 ± 2.4; P = 0.000092	WT = 6.3 ± 4.9; PAFR$^{-/-}$ = 102.7 ± 119.5
**22 Hz**	WT = 0.8 ± 0.2; PAFR$^{-/-}$ = 0.3 ± 0.1; P = 0.000179	WT = -44.3 ± 14.1; PAFR$^{-/-}$ = -88.4 ± 67.7
**26Hz**	WT = 0.7 ± 0.2; PAFR$^{-/-}$ = 0.4 ± 0.1; P = 0.014874	WT = -79.6 ± 15.9; PAFR$^{-/-}$ = -57.2 ± 16.9; P = 0.041979
**30 Hz**	WT = 0.5 ± 0.1; PAFR$^{-/-}$ = 0.2 ± 0.1; P = 0.000089	WT = -129.0 ± 13.1; PAFR$^{-/-}$ = -100.3 ± 27.3

ON/OFF ERG		
	**Amplitude**	**Phase**
**Mesopic ON**	WT = 55.7 ± 8.7; PAFR$^{-/-}$ = 34.5 ± 13.3; P = 0.0005	WT = -123.8 ± 6.8; PAFR$^{-/-}$ = -117.0 ± 12.5 P = 0.03
**Mesopic OFF**	WT = 43.9 ± 7.7; PAFR$^{-/-}$ = 28.3 ± 9.9; P = 0.000015	WT = 46.9 ± 6.4; PAFR$^{-/-}$ = 61.0 ± 12.3
**Photopic ON**	WT = 16.9 ± 1.7; PAFR$^{-/-}$ = 7.5 ± 2.8; P = 0.000573	WT = -116.3 ± 5.2.; PAFR$^{-/-}$ = -128.1 ± 28.1 P = 0,03
**Photopic OFF**	WT = 13.8 ± 1.9; PAFR$^{-/-}$ = 4.6 ± 2.0; P = 0,00015	WT = 61.35 ± 4.7; PAFR$^{-/-}$ = 55.1 ± 35.0
